# Epidemiology, Clinical Characteristics and Outcomes of Extensively Drug-Resistant *Acinetobacter baumannii* Infections among Solid Organ Transplant Recipients

**DOI:** 10.1371/journal.pone.0052349

**Published:** 2012-12-20

**Authors:** Ryan K. Shields, Cornelius J. Clancy, Louise M. Gillis, Eun J. Kwak, Fernanda P. Silveira, Rima C. Abdel Massih, Gregory A. Eschenauer, Brian A. Potoski, M. Hong Nguyen

**Affiliations:** 1 Department of Medicine, University of Pittsburgh, Pittsburgh, Pennsylvania, United States of America; 2 Antibiotic Management Program, University of Pittsburgh Medical Center, Pittsburgh, Pennsylvania, United States of America; 3 VA Healthcare System Pittsburgh, Pittsburgh, Pennsylvania, United States of America; 4 Department of Pharmacy & Therapeutics, University of Pittsburgh, Pittsburgh, Pennsylvania, United States of America; University of Calgary, Canada

## Abstract

**Background:**

Extensively drug-resistant *Acinetobacter baumannii* (XDR-*Ab*) has emerged as a major nosocomial pathogen, but optimal treatment regimens are unknown. Although solid organ transplant (SOT) recipients are particularly susceptible to XDR-*Ab* infections, studies in this population are limited. Our objectives were to determine the epidemiology, clinical characteristics and outcomes of XDR-*Ab* infections among SOT patients.

**Methods:**

A retrospective study of SOT recipients at our center who were colonized or infected with XDR-*Ab* between November 2006 and December 2011 was conducted. Among infected patients, the primary outcome was survival at 28 days. Secondary outcomes included survival at 90 days and clinical success at 28 days, and XDR-*Ab* infection recurrence.

**Results:**

XDR-*Ab* was isolated from 69 SOT patients, of whom 41% (28) and 59% (41) were colonized and infected, respectively. Infections were significantly more common among cardiothoracic than abdominal transplant recipients (p = 0.0004). Ninety-eight percent (40/41) of patients had respiratory tract infections, most commonly ventilator-associated pneumonia (VAP; 88% [36/41]). Survival rates at 28 and 90 days were 54% (22/41) and 46% (19/41), respectively. Treatment with a colistin-carbapenem regimen was an independent predictor of 28-day survival (*p* = 0.01; odds ratio = 7.88 [95% CI: 1.60–38.76]). Clinical success at 28 days was achieved in 49% (18/37) of patients who received antimicrobial therapy, but 44% (8/18) of successes were associated with infection recurrence within 3 months. Colistin resistance emerged in 18% (2/11) and 100% (3/3) of patients treated with colistin-carbapenem and colistin-tigecycline, respectively (*p = *0.03).

**Conclusions:**

XDR-*Ab* causes VAP and other respiratory infections following SOT that are associated with significant recurrence and mortality rates. Cardiothoracic transplant recipients are at greatest risk. Results from this retrospective study suggest that colistin-carbapenem combinations may result in improved clinical responses and survival compared to other regimens and may also limit the emergence of colistin resistance.

## Introduction

Bacteria cause most infections following solid organ transplantation (SOT) and bacterial infections are a leading cause of death across transplant types [1], [2]. Infections due to bacteria that are resistant to multiple antimicrobial agents pose an increasing threat to SOT recipients and other hospitalized patients [3], [4]. Indeed, SOT recipients are highly susceptible to the acquisition or development of drug-resistant bacterial infections due to their frequent and prolonged hospitalizations, receipt of broad-spectrum antibiotics, multiple surgeries and concomitant immunosupression [5]. *Acinetobacter baumannii* has emerged in recent years as a particularly problematic drug-resistant pathogen [6], [7], but it has been rarely studied among SOT patients [4], [5], [8], [9].

*A. baumannii* is a resilient opportunistic pathogen that is notorious for persisting in the hospital environment. It is intrinsically resistant to some beta-lactam antibiotics, but is better known for its ability to acquire resistance to all commercially available antimicrobial agents [10]. In fact, carbapenem-resistant *A. baumannii* has been identified as one of the six “ESKAPE” pathogens responsible for an increasing number of nosocomial infections in the United States [3]. Carbapenem-resistant *A. baumannii* isolates are almost always extensively drug-resistant (XDR), which is defined by resistance to all antimicrobial agents except polymixins (colistin) and tigecyline [11]. The treatment of XDR-*A. baumannii* (XDR-*Ab*) infections is a major challenge given the lack of effective treatment options and limited management experience. Unfortunately, increased use of colistin and tigecycline as salvage therapy has been associated with the emergence of pan-drug resistant (PDR)-*Ab*, against which no known antimicrobial agents retain activity [11].

In the SOT program at the University of Pittsburgh Medical Center (UPMC), we cared for several patients with serious XDR-*Ab* infections who failed to respond to various combinations of antibiotics that included colistin and/or tigecycline. In a pilot study initiated in 2009, our Antimicrobial Management and Transplant Infectious Diseases (AMP and TID) programs collaborated to devise an institution-specific treatment regimen for XDR-*Ab* infections [4]. We used a variety of methods to test XDR-*Ab* isolates recovered from our patients for *in vitro* susceptibility to antimicrobial combinations. On the basis of our results, we recommended that the combination of colistin and a carbapenem be adopted as standard therapy for XDR-*Ab* infections among SOT patients at our center. We reported encouraging short-term clinical success and survival rates among five SOT recipients treated with this regimen [4]. To our knowledge, this was the first study to assess the efficacy of a colistin-carbapenem combination regimen in the treatment of XDR-*Ab* infections.

The present study is a follow-up to our preliminary report, in which we describe the epidemiology, clinical characteristics s and long-term outcomes of SOT recipients with XDR-*Ab* infections. In so doing, we report survival, treatment responses and the emergence of colistin resistance among patients treated with colistin-carbapenem and other regimens.

## Methods

### Ethics Statement

The protocol was reviewed by the University of Pittsburgh Institutional Review Board (IRB) and determined to meet the necessary criteria for exemption under section 45 of the Code of Federal Regulations. Per local policies and through consultation with the IRB, written patient consent was not required and formal ethical approval was reviewed and waived.

### Patient Population and Definitions

We conducted a retrospective study of all 69 SOT recipients at UPMC who were colonized or infected with XDR- or PDR*-Ab* between November 2006 and December 2011. Sixteen of these 69 patients were included in our earlier study that identified the combination of a carbapenem and colistin as the most active regimen against XDR-*Ab* isolates from our center [4]. XDR and PDR-*Ab* was defined according to consensus recommendations [11]. All patients were evaluated and managed by our TID service as part of routine patient care. Classifications of colonization or infection (disease caused by *Ab*) were made by the treating TID physician and independently confirmed by two investigators according to consensus criteria [12]. Patients with clinical evidence of infection were classified as having pneumonia [ventilator-associated (VAP) or healthcare-associated (HAP)], tracheobronchitis [ventilator-associated (VAT)] or bacteremia [13].

### Patient Outcomes

The primary outcome of our analysis was survival at 28 days following the onset of XDR-*Ab* infection. Twenty-eight rather than 14 day survival was used because many patients remained on antimicrobial therapy at the 14 day endpoint. Moreover, the 28 day endpoint allowed for a more reasonable definition of recurrent infection (see below). Secondary outcomes included survival at 90 days and in-hospital. Persistent XDR-*Ab* infection at the time of death was defined as a positive culture on autopsy or within 7 days of death. Clinical success was evaluated at 28 days from the onset of infection and defined as the improvement or resolution in signs and symptoms of infection [4]. Patients with clinical success at 28 days who manifested new signs and symptoms of infection due to XDR-*Ab* within the following 3 months were classified as having recurrent infections.

### Colistin Susceptibility Testing

Colistin susceptibility testing was performed by our clinical microbiology laboratory by broth dilution methods at the request of the treating physician [14]. Susceptible isolates were defined by a colistin MIC≤2 µg/mL.

### Statistical Analysis

Comparisons between two groups were performed by Wilcoxon rank sum test for continuous variables and Chi-squared or Fisher’s exact tests for categorical variables. Multivariate logistic regression analysis was performed by backward selection procedures. We included variables identified (p-value<0.20) by univariate analyses. Kaplan-Meier survival analysis was used to compare survival between treatment groups. Significance was defined as p-value≤0.05 (two-tailed).

## Results

### XDR-*A. baumannii* (XDR-*Ab*) Colonization and Infections

XDR-*Ab* was isolated from 69 patients following lung (36%; 25/69), liver (25%; 17/69), kidney (17%; 12/69), heart (9%; 6/69), multivisceral (6%; 4/69), intestine (4%; 3/69) or pancreas (3%; 2/69) transplantation. Forty-one percent (28/69) and 59% (41/69) of patients were colonized and infected, respectively. Rates of colonization and infection by transplant type are presented in Table 1. XDR-*Ab* infections were more common among cardiothoracic transplant (heart and lung) than abdominal transplant (intestine, kidney, liver, multivisceral, and pancreas) recipients (2.6% [24/901] *vs.* 0.9% [17/1971]; *p = *0.0004).

**Table 1:** Colonization and infection by transplant type.

Transplant Type	All Transplant Patients	XDR-Ab Colonization n (%)	XDR-Ab Infection n (%)
Heart	272	0 (0)	6 (2.2)
Intestine	75	2 (2.7)	1 (1.3)
Kidney	1001	5 (0.5)	7 (0.7)
Liver	691	10 (1.4)	7 (1)
Lung*	653	7 (1.1)	18 (2.8)
Multivisceral†	153	3 (2)	1 (0.7)
Pancreas	51	1 (2)	1 (2)
Total	2896	28 (0.9)	41 (1.4)

n = number.

* Includes patients with simultaneous heart and lung transplant. One lung transplant patient was infected with PDR-Ab.

† Multivisceral includes patients with simultaneous liver/intestine (2), liver/kidney (1), and liver/intestine/pancreas/stomach (1) transplantation.

Median times to colonization and infection post-transplant were 121 and 172 days, respectively (ranges: 0–2795 and 0–6829 days, respectively; *p = *0.82). The sites of colonization were the respiratory tract (53%; 15/28), urine (36%; 10/28), and wounds (11%; 3/28). In contrast, the respiratory tract was the site of XDR-*Ab* infection in 98% (40/41) of patients.

### Demographics and Clinical Characteristics of Patients with XDR-*Ab* Infections

Thirty-two percent (13/41) of patients with an XDR-*Ab* infection were previously colonized. Infected patients were critically-ill at the time of diagnosis, as evident by APACHE II scores and high rates of ICU stay and mechanical ventilation (Table 2). Ninety percent (37/41) and 7% (3/41) of infected patients had pneumonia (VAP = 36 and HAP = 1) and VAT, respectively. The only patient who did not have a respiratory tract infection had primary bacteremia. Twenty-five percent (9/36) of VAP was complicated by associated infections, including secondary bacteremia (n = 5), mediastinitis (n = 2), and empyema (n = 2). Bacterial co-pathogens were identified in 44% (18/41) of patients, including carbapenem-resistant co-pathogens in 10% (4/41).

**Table 2:** Clinical characteristics and demographics of patients with XDR-*Ab* infection.

Clinical Characteristic	XDR-*Ab* Infection (n = 41)
Median age, years (range)	56 (21–80)
Male, no. (%)	26 (63%)
Caucasian, no. (%)	35 (85%)
Median time from transplant, days (range)	172 (0–6829)
Median APACHE II (range)	19 (8–29)
Prior XDR-*Ab* colonization, no. (%)	13 (32%)
Co-pathogen present, no. (%)*	18 (44%)
Intensive care unit, no. (%)†	40 (98%)
Mechanical ventilation, no. (%)†	40 (98%)
Renal replacement therapy, no. (%)†	16 (39%)

* Co-pathogens included: *Pseudomonas* (8), *Enterobacter* (3), *Serratia* (3), *Stenotrophomonas* (1), *Klebsiella* (1), *Pantoea* (1), and methicillin-resistant *Staphylococcus aureus* (1).

† At the time of disease onset.

The 28 day survival rate among infected patients was 54% (22/41). The 90 day and in-hospital survival rates were 46% (19/41) and 39% (16/41), respectively. Among patients who died in-hospital, 68% (17/25) had persistent XDR*-Ab* infection at the time of death.

### Patient Outcomes by Treatment Regimen

**Figure 1:**
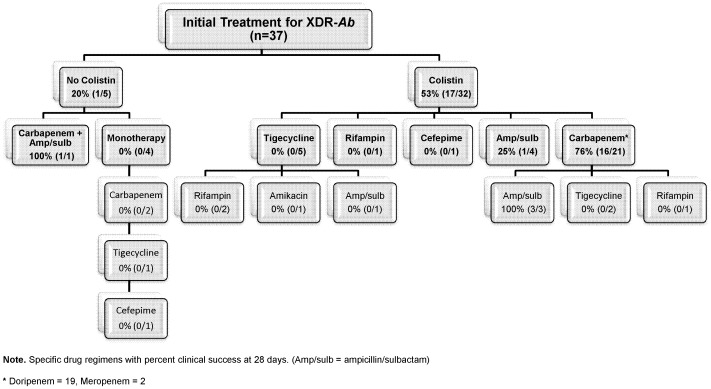
This figure provides a breakdown of each specific treatment regimen used among 37 solid-organ transplant recipients with XDR-Ab infections. The rates of 28-day clinical success for each regimen are listed.

**Figure 2:**
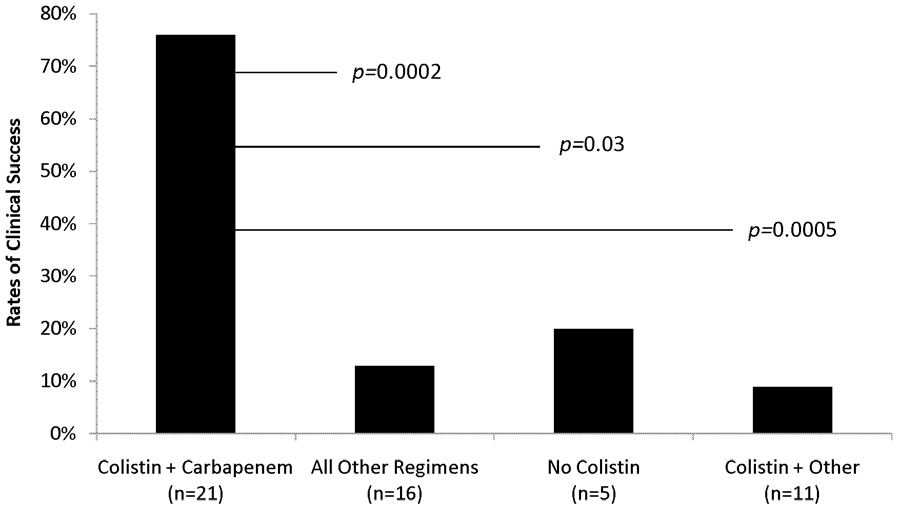
This figure provides a graphical representation of 28-day clinical success rates and compares colistin-carbapenem regimens to other treatment regimens.

Ten percent (4/41) of infected patients died prior to the institution of antimicrobial therapy. Among the remaining 90% (37/41) of patients who were treated with antimicrobials against XDR-*Ab*, the clinical success rate at 28 days was 49% (18/37). Clinical success rates for specific antimicrobial regimens are summarized in Figures 1 and 2. Eleven percent (4/37) and 89% (33/37) of patients received mono- and combination therapy, respectively. The median durations of mono- and combination therapy were 6.5 (range: 5–8) and 15 (range: 3–52) days, respectively (*p = *0.13). Clinical success was not achieved for any patients receiving monotherapy (0/4). Ninety-seven percent (32/33) of combination regimens were colistin-based and 60% (21/32) of colistin-based combinations included a carbapenem (doripenem, *n = *19, and meropenem, *n = *2; Figures 1 and 2). Clinical characteristics of patients receiving colistin and carbapenem combination therapy were similar to patients treated with other regimens (Table 3). Rates of clinical success at 28 days were significantly higher when colistin was combined with a carbapenem (76%; 16/21) versus other agents (9% [1/11]; *p* = 0.0005, Figure 2). None of the 7 patients receiving colistin and tigecycline experienced clinical success. Clinical success at 28 days was similar for patients who received inhaled colistin as an adjunct to combination therapy (57%; 8/14) versus those who did not (50%; 9/18) (*p = *0.73).

Forty-four percent (8/18) of patients with clinical success at 28 days had recurrent infections. The initial infections in these patients were VAP (n = 7) and VAT (n = 1). All patients with recurrent infections initially received colistin-carbapenem combination therapy for a median duration of 14 days (range: 5–28). The recurrent disease manifestations were VAP (n = 7) and bacteremia (n = 1). Twenty-eight percent (5/18) of patients with clinical success at 28 days had multiple recurrences. Patients with recurrent infections were more likely to be colonized with XDR-*Ab* prior to the onset of infection and had significantly longer hospital stays compared to patients with no recurrences (*p* = 0.04 and 0.02, respectively; Table 4). In all instances, recurrent infections were re-treated with colistin-carbapenem combinations. Ultimately, clinical success was achieved in 75% (6/8) of patients (Table 5). Ampicillin/sulbactam was added to the colistin-carbapenem regimen in 50% (3/6) of successfully-treated recurrent infections.

**Table 3:** Disease severity and adjunctive treatment by regimen.

Clinical Characteristic	Colistin+Carbapenem (n = 21)	Other Regimens (n = 16)	p-value
Median APACHE II score (range)	19 (10–29)	20 (8–28)	0.93
Co-pathogen present, no. (%)	9 (43%)	9 (56%)	0.52
Intensive care unit, no. (%)	20 (95%)	20 (100%)	1.00
Mechanical ventilation, no. (%)	20 (95%)	20 (100%)	1.00
Ventilator-associated pneumonia, no. (%)	17 (81%)	15 (94%)	0.36
Associated infection, no. (%)	3 (14%)	5 (31%)	0.25
Inhaled colistin, no. (%)*	10 (48%)	5 (31%)	0.50
Median duration of treatment, days (range)	15 (5–44)	7.5 (3–52)	0.09

* Only patients with XDR-*Ab* pneumonia received inhaled colistin; all patients receiving inhaled colistin were on concomitant systemic colistin therapy.

**Table 4:** Clinical characteristics of patients with and without recurrence of XDR-*Ab* infection.

Clinical Characteristic	Recurrence (n = 8)	No Recurrence (n = 8)	p-value
Median duration of combination therapy, days (range)	14 (5–28)	21.5 (7–44)	0.27
Median APACHE score (range)	19 (12–27)	21 (15–29)	0.37
Extubation, no (%)†	2 (25)	5 (63)	0.31
VAP as first infection, no. (%)	7 (88)	5 (63)	0.57
Mechanical ventilation at EOT, no. (%)	6 (75)	5 (63)	1.00
Ampicillin/sulbactam as initial therapy, no. (%)	0 (0)	3 (38)	0.20
Prior XDR-*Ab* colonization, no. (%)	6 (75)	1 (13)	0.04
Co-pathogen present, no. (%)	5 (63)	2 (25)	0.31
Inhaled colistin, no. (%)	3 (38)	5 (63)	0.62
Median length of stay from disease onset, days (range)*	94 (21–232)	29 (15–80)	0.02

EOT = end of treatment; VAP = ventilator-associated pneumonia.

† Extubated (ET tube removed) at the time of recurrence, 90 days, or death (whichever occurred first).

* Days from the onset of initial infection to discharge or death.

**Table 5:** Characteristics of Recurrent XDR-*Ab* Infections.

Subject	InitialDisease	Rx (Duration, days)	FirstRecurrence	Rx (Duration, days)	Outcome	# of Additional Recurrences	FinalRecurrence	Rx (Duration, days)	Outcome	MicroEradication	Final Outcome
1	VAP	CD (28)	VAP	CD (28)	Success	0	–	–	–	Yes	Death 232 days after infection^1^
2	VAT	CD (5)	VAP	CDA (18)	Success, Recurrence	3	VAP	CDA (71)	Success	Yes	Death 250 days after infection^2^
3	VAP	CD (14)	VAP	CD (17)	Success, Recurrence	1	VAP	CDA (22)	Success	Yes	Alive
4	VAP	CD (21)	VAP	CD (14)	Success, Recurrence	1	VAP	No Rx^3^	Death	No	Death
5	VAP	CD (13)	VAP	CD (10)	Success, Recurrence	3	VAP	CDA (3)	Death	No	Death
6	VAP	CD (14)	VAP	CD (15)	Failure	1	VAP	CDA (15)	Success	Yes	Alive
7	VAP	CD (14)	Bacteremia	CD (10)	Success	0	–	–	–	Yes	Alive
8	VAP	CD (7)	VAP	CD (7)	Success	0	–	–	–	Yes	Death 107 days after infection^4^

A = Ampicillin/sulbactam; C = Colistin; D = Doripenem; VAP = Ventilator-associated pneumonia; Rx = Antimicrobial Therapy.

1 Death due to primary graft failure (intestine).

2 Death due to *Pseudomonas* pneumonia.

3 Patient died prior to the initiation of treatment.

4 Death due to chronic respiratory failure.

**Figure 3:**
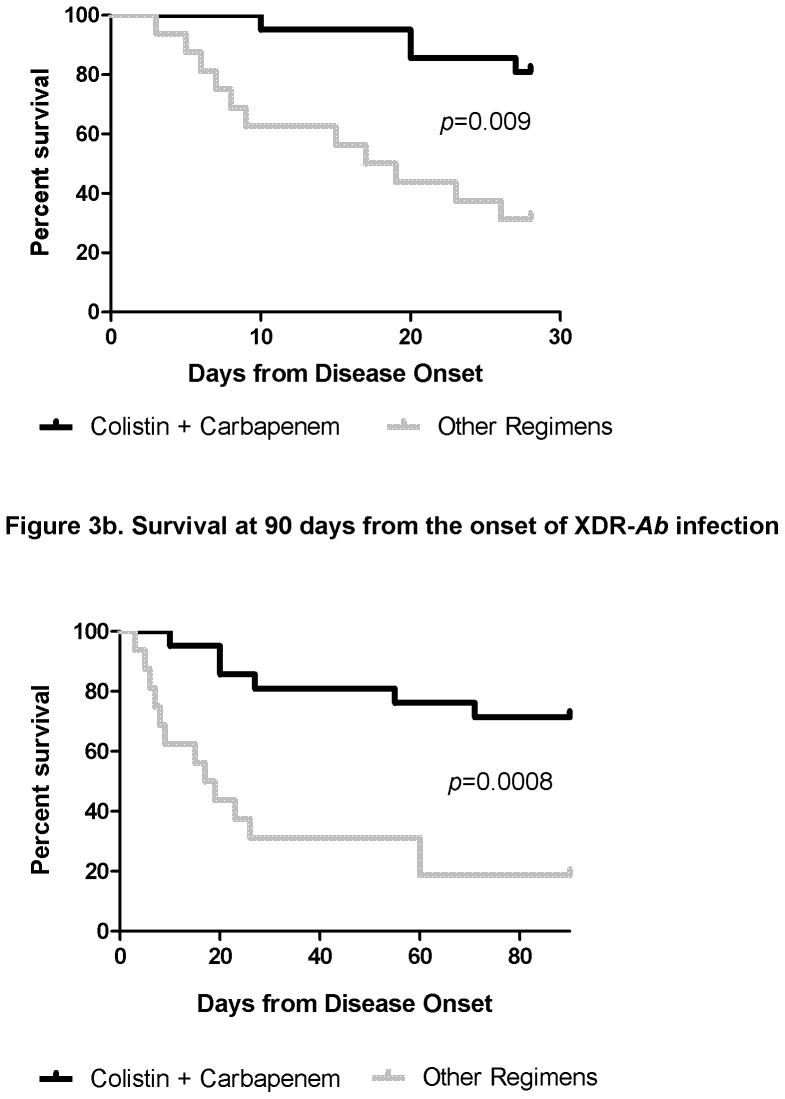
**Figures 3a and 3b** provide Kaplan-Meier curves comparing 28 and 90-day survival, respectively. The graphs compare colistin-carbapenem regimens to other treatment regimens.

The 28 day survival rate for patients treated with antimicrobials was 59% (22/37). None of the patients (0/4) who were treated with antimicrobial monotherapy survived for 28 days, compared to 66% (22/33) of patients receiving combination therapy (*p = *0.03). The 90 day and in-hospital survival rates for the latter patients were 62% (23/37) and 49% (18/37), respectively. Colistin-carbapenem regimens were associated with significantly higher 28 and 90 day survival compared to other regimens (*p = *0.009 and 0.0008, respectively; Table 6, Figures 3a, 3b). Moreover, colistin-carbapenem regimens were independently associated with 28 day survival by multivariate logistic regression (Table 7; *p = *0.01, Odds ratio = 7.88 [95% CI: 1.60–38.76]). Long-term survival at 90 days was significantly higher for patients who had clinical success at 28 days (95% [18/19]) compared to those without clinical success (6% [1/18], *p<*0.0001).

**Table 6:** Mortality rates by treatment type.

Mortality at:	No Treatment (n = 4) (%)	No Colistin (n = 5) (%)	Colistin+Carbapenem(n = 21) (%)	Colistin+Other (n = 11) (%)
14 days	4 (100)	3 (60)	1 (5)	3 (27)
28 days	4 (100)	4 (80)	4 (19)	7 (64)
90 days	4 (100)	4 (80)	5 (24)	9 (82)
In-hospital	4 (100)	2* (40)	9 (43)	10 (91)

* Two patients were discharged to hospice prior to death and are not counted as in-hospital death.

**Table 7:** Predictors of 28 day survival among patients treated for XDR-*Ab* infections.

Clinical Characteristic	28 Day Survival		Univariate p-value	Multivariate p-value
	Alive (n = 22)	Dead (n = 15)		
Median age, years (range)	57.5 (36–77)	58 (21–80)	0.62	
Male, no. (%)	13 (59)	10 (67)	0.74	
Caucasian, no. (%)	20 (91)	12 (80)	0.38	
Median time from transplant, days (range)	128.5 (0–3781)	202 (0–6829)	0.68	
Cardiothoracic transplant, no. (%)	12 (55)	9 (60)	1.00	
Prior XDR-*Ab* colonization, no. (%)	10 (45)	3 (20)	0.17	0.16
Median APACHE II score (range)	19.5 (8–29)	19 (10–28)	0.73	
Co-pathogen present, no. (%)†	11 (50)	7 (47)	1.00	
Intensive care unit, no. (%)†	21 (95)	15 (100)	1.00	
Mechanical ventilation, no. (%)†	21 (95)	15 (100)	1.00	
Ventilator-associated pneumonia, no. (%)†	18 (82)	14 (93)	0.63	
Renal replacement therapy, no. (%)†	7 (32)	6 (40)	0.73	
Colistin *in vitro* resistance*	5% (1/19)	0% (0/9)	1.00	
Inhaled colistin, no. (%)	11 (50)	4 (27)	0.19	0.20
Colistin+carbapenem therapy, no. (%)	17 (77)	4 (27)	0.006	0.01

† At the time of disease onset.

* Defined as a colistin MIC>2 µg/mL. Twenty-eight patients received a colistin-containing regimen and had colistin susceptibility test results available.

### Emergence of Colistin Resistance

Colistin MICs were determined against 88% (28/32) of XDR*-Ab* isolates recovered from patients at the time of initial treatment with a colistin-containing regimen. The median colistin MIC was 0.5 µg/mL (range: 0.125–8 µg/mL); 4% (1/28) of patients were infected with a colistin-resistant isolate (MIC = 8 µg/mL; PDR-*Ab*). There were no differences in median colistin MICs among patients who survived or died at 28 days (0.25 µg/mL (range: 0.125–2 µg/mL) *vs.* 0.125 µg/mL (range: 0.125–8 µg/mL); p = 0.15). Isolates were available for repeat susceptibility testing in 50% (14/28) of patients at a median of 46 days (range: 7–124 days) after the initial MIC was measured. Colistin resistance emerged in 36% (5/14; Tables 8 and 9). There were no differences in the duration of inhaled (*p = *0.19) or intravenous (*p = *0.24) colistin therapy among patients who did and did not develop resistance. One hundred percent (3/3) of isolates collected from patients who received colistin-tigecycline combinations developed resistance compared to 18% (2/11) of isolates from patients who received colistin and a carbapenem (*p* = 0.03).

**Table 8:** Development of colistin resistance among XDR-*Ab* isolates.

Subject	Initial colistin MIC (µg/mL)	Repeat colistin MIC (µg/mL)	Days between MICs	Days of Inhaled Colistin	Days ofIntravenous Colistin	Treatment Regimen
1	0.125	16	124	32	42	Colistin/Doripenem
2	0.125	64	16	0	14	Colistin/Tigecycline
3	0.5	256	55	51	39	Colistin/Tigecycline
4	0.125	32	91	88	67	Colistin/Tigecycline
5	1	64	23	23	23	Colistin/Doripenem
Median (Range)	0.125 (0.125–1)	64 (16–256)	55 (16–124)	32 (0–88)	39 (14–67)	

**Table 9:** Absence of colistin resistance among XDR-*Ab* isolates.

Subject	Initial colistinMIC (µg/mL)	Repeat colistinMIC (µg/mL)	Days between MICs	Days of Inhaled Colistin	Days of Intravenous Colistin	Treatment Regimen
6	0.5	0.5	86	0	15	Colistin/Doripenem
7	2	0.5	62	24	22	Colistin/Doripenem
8	0.125	1	23	0	16	Colistin/Doripenem
9	2	0.125	42	6	31	Colistin/Doripenem
10	1	0.5	25	0	19	Colistin/Doripenem
11	0.5	2	7	0	6	Colistin/Meropenem
12	0.25	0.25	28	25	25	Colistin/Doripenem
13	0.5	0.5	50	0	18	Colistin/Doripenem
14	2	0.25	107	107	65	Colistin/Doripenem
Median(Range)	0.5 (0.125–2)	0.5 (0.125–2)	42 (7–107)	0 (0–107)	19 (6–65)	

## Discussion

To our knowledge, this is the largest study describing the epidemiology, clinical manifestations, and outcomes of XDR-*Ab* infections among SOT recipients. Over a five year period, we identified 41 SOT patients who developed invasive XDR-*Ab* infections. Among 37 patients treated with antimicrobial regimens against XDR-*Ab,* the rates of clinical success and survival at 28 days were 49% (18/37) and 57% (21/37), respectively. The major finding of this study was the improved outcomes among patients treated with colistin-carbapenem combination therapy compared to other therapeutic regimens. In fact, clinical success and survival at 28 days was achieved in 76% (16/21) and 81% (17/21) of patients receiving colistin and a carbapenem compared to 13% (2/16) and 31% (5/16), respectively, for patients who received other regimens. Treatment with a colistin-carbapenem regimen was an independent predictor of 28-day survival. Colistin-carbapenem therapy also limited the emergence of colistin resistance when compared to treatment with colistin-tigecycline. Taken together, our results corroborate and extend those of an earlier pilot study, in which we reported 28 day clinical success rates of 80% (4/5) and 9% (1/11) among SOT patients who received colistin-carbapenem or other regimens, respectively, for the treatment of XDR-*Ab* [4].

These data are particularly important given the lack of therapeutic options against XDR-*Ab* infections. By definition, XDR-*Ab* isolates are resistant to all conventional antimicrobial agents. Moreover, treatment of patients with potential salvage agents such as tigecycline and colistin has been associated with poor clinical outcomes including death, breakthrough infections, and the emergence of PDR-*Ab* infections [15], [16], [17]. As a result of the dismal experience to date, investigators and clinicians have explored a variety of drug combinations in the laboratory and clinic. An increasing number of studies have demonstrated positive *in vitro* interactions between various combinations of carbapenems, colistin, rifampin, sulbactam, and tigecycline against XDR-*Ab* isolates [18], [19], [20], [21], [22], [23], [24], [25], [26], [27], [28], [29], [30], [31], [32]. Nevertheless, clinical studies validating *in vitro* findings are limited. Most experience in patients has been reported for combination therapy with colistin plus rifampin [33], [34], a carbapenem [4], [35], [36] or tigecycline [37], but clinical success rates are highly variable and long-term outcomes of patients are unknown. In this regard, our study is unique not only for its size and the high rates of clinical success achieved with colistin-carbapenem combination therapy, but also for the insights it provides into long term outcomes such as the development of colistin resistance and XDR-*Ab* disease recurrence. In fact, all surviving patients in our study were followed for a minimum of 6 months from the onset of XDR-*Ab* infection, an observation period that was largely made possible by prolonged survival achieved with colistin-carbapenem combinations.

To our knowledge, this is the first report to demonstrate that the use of colistin-carbapenem combination therapy in a clinical setting may limit the emergence of colistin resistance. Our data for serial XDR-*Ab* isolates recovered from patients treated with colistin-containing combinations are consistent with prior *in vitro* pharmacokinetic/pharmacodynamic analyses of colistin and doripenem against *K. pneumoniae* isolates [38]. It is increasingly clear that colistin monotherapy is untenable due to the frequent emergence of colistin resistance among *Acinetobacter* isolates, which correlates with therapeutic failures and stems from point mutations and/or the deletion of genes for outer membrane lipopolysaccharides [39]. Therefore, colistin-carbapenem therapy may provide the dual benefit of improving clinical outcomes among patients with XDR-*Ab* infections and suppressing the emergence of further drug resistance. The use of multiple antimicrobials to limit drug resistance has long been cited as a rationale for employing combination regimens to treat infectious diseases. Clinical data validating this strategy are well-established for infections caused by *Mycobacterium tuberculosis* [40] and human immunodeficiency virus [41], [42], [43], but evidence during the treatment of infections by Gram negative bacteria are limited [44].

Despite the improved outcomes we observed among patients treated with colistin-carbapenem, it is important to acknowledge that clinical responses were still suboptimal. Indeed, 50% (8/16) of patients who were successfully treated with the regimen at 28 days subsequently had recurrent XDR-*Ab* infections, including 31% (5/16) who suffered multiple recurrences. Therefore, initial colistin-carbapenem regimens were associated with long-term cures, recurrent infections and clinical failure in 38% (8/21), 38% (8/21) and 24% (5/21) of patients, respectively. Fortunately, 75% (6/8) of patients with recurrent infections were ultimately cured with repeated colistin-carbapenem regimens, often including the addition of sulbactam. While the long-term outcomes were encouraging, the treatment of recurrent infections resulted in significantly longer hospital stays. Due to the lack of sufficient follow-up, our pilot study and other reports of patient outcomes did not assess recurrent XDR-Ab infections [4],[33],[34],[35],[36],[37]. Our long-term experience is not surprising, as persistence of *Acinetobacter* is well-recognized, especially among immune compromised individuals [8]. In one report, for example, *Ab* was repeatedly recovered from respiratory cultures in 67% of transplant recipients compared to only 7% of non-transplant patients (*p = *0.005) [8]. The ability of *Ab* to persist within human hosts is likely multi-factorial. Of course, the ease with which *Ab* acquires a wide variety of drug resistance mechanisms facilitates survival despite aggressive antimicrobial therapy. In addition, the organism is particularly well-adapted to adhere to human epithelial cells and withstand adverse environmental conditions due to its innate resistance to desiccation and disinfectants [45]–[46]. Indeed, *Ab* forms biofilms on human cells and abiotic surfaces, such as glass or plastic, allowing it to survive for months in the face of dry conditions or nutrient starvation [46]. As such, it is unlikely that any treatment regimen will completely eliminate recurrent XDR-*Ab* infections, and preventive strategies, in particular scrupulous infection control measures and judicious use of antibiotics, are crucial to improving patient outcomes [47].

The importance of prevention is highlighted by our findings that 29% of infected patients were known to be colonized previously with XDR-*Ab* and recurrent infections were more common among colonized patients. The overall colonization and infection rates among almost 3,000 SOT recipients were 0.9% and 1.4%, respectively. Interestingly, colonization rates were similar for all types of transplant, but invasive infections were more likely among cardiothoracic transplant recipients. XDR-*Ab* almost exclusively caused respiratory infections in our study, consistent with previous reports among SOT patients [5], [8]. The vast majority of infections were VAP, in keeping with the fact that 98% of patients were in the ICU and intubated at the onset of infection. *Ab* utilizes short, fimbrial-like protrusions on the bacterial cell surface to attach with great avidity to bronchial epithelial cells [48], [49]. Lung transplant recipients, in particular, are predisposed to respiratory tract infections by *Ab* and other pathogens because of their need for prolonged ventilation [50], impaired mucociliary clearance [51], and profound immune suppression [52]. Given the severity of pneumonia among immune comprised hosts, it is not surprising that 61% of our patients died in the hospital, the majority with ongoing XDR-*Ab* infections. Mortality rates of *Ab* infections among SOT recipients in other reports have ranged from 50–80% [5], [8], [9], which exceed those in non-transplant patients with similar diseases and for VAP due to other pathogens [16], [53], [54].

There are several limitations to our study, including the fact that it is a single center experience that may not be representative of other institutions. As for all retrospective studies, data were limited to existing medical records and information such as prior antimicrobial therapy was not necessarily available for all patients. Our retrospective study design is subject to selection bias and management decisions for patients were uncontrolled. As such, we cannot draw definitive conclusions when comparing treatment regimens. Nevertheless, the underlying diseases, clinical parameters and adjunctive therapies for patients receiving colistin/carbapenem combinations were similar to patients treated with other agents. The odds ratio for 28 day survival among patients receiving colistin/carbapenem regimens was highly significant (OR = 7.88, *p = *0.01), but the associated confidence interval was wide due to our relatively small sample sizes. All patients were managed by the TID and AMP teams, but antimicrobial dosing was not standardized during the study period, and doripenem and colistin MICs were not requested for all isolates. Of note, doripenem was used as the preferred carbapenem at our center, but *in vitro* data from our lab and others suggest that other anti-pseudomonal carbapenems may be equally efficacious [4], [31], [55].

The colistin-carbapenem regimen was initially recommended as frontline therapy against XDR-*Ab* at our center based on *in vitro* data from our AMP lab. Due to the poor outcomes we had observed among XDR-*Ab*-infected patients, we systematically tested isolates from our patients in our pilot study for susceptibility to various antimicrobial combinations using time-kill assays and other methods. Colistin-carbapenem was identified as the most promising combination because it commonly showed positive interactions *in vitro* and colistin MICs against baseline patient isolates were generally in the susceptible range. In this regard, the present study suggests a model by which centers may employ local susceptibility and combination testing data to identify or corroborate optimal treatment regimens against resistant pathogens. Given the well-recognized heterogeneity of *Ab* isolates from different centers and geographic locations [25], [47], [56], it is important to recognize that the most effective treatment regimens may vary. In assessing our experience, it is also important to consider that our studies were conducted among SOT recipients, a patient population that is generally excluded from clinical trials of antimicrobial therapy. In fact, we propose that SOT and other immune-compromised patients may represent promising populations for studying the treatment of resistant bacterial infections, as they are particularly dependent upon antimicrobial efficacy for positive clinical outcomes. In these patients, antimicrobial effects are likely to be most relevant for serious infections such as VAP, which are associated with very high microbial burdens.
